# Overwintering Is Associated with Reduced Expression of Immune Genes and Higher Susceptibility to Virus Infection in Honey Bees

**DOI:** 10.1371/journal.pone.0129956

**Published:** 2015-06-29

**Authors:** Nadja Steinmann, Miguel Corona, Peter Neumann, Benjamin Dainat

**Affiliations:** 1 Agroscope—Swiss Bee Research Center—Liebefeld, Schwarzenburgstrasse 161, 3003 Bern, Switzerland; 2 Institute of Bee Health, Vetsuisse Faculty and Faculty of Medicine, University of Bern, Bremgartenstr. 109a, 3001 Bern, Switzerland; 3 Bee Research Laboratory USDA-ARS, Beltsville, MD 20705, United States of America; 4 Swiss Bee Health Service, apiservice, Schwarzenburgstrasse 161, 3003 Bern, Switzerland; Salford University, UNITED KINGDOM

## Abstract

The eusocial honey bee, *Apis mellifera*, has evolved remarkable abilities to survive extreme seasonal differences in temperature and availability of resources by dividing the worker caste into two groups that differ in physiology and lifespan: summer and winter bees. Most of the recent major losses of managed honey bee colonies occur during the winter, suggesting that winter bees may have compromised immune function and higher susceptibility to diseases. We tested this hypothesis by comparing the expression of eight immune genes and naturally occurring infection levels of deformed wing virus (DWV), one of the most widespread viruses in *A*. *mellifera* populations, between summer and winter bees. Possible interactions between immune response and physiological activity were tested by measuring the expression of *vitellogenin* and methyl farnesoate epoxidase, a gene coding for the last enzyme involved in juvenile hormone biosynthesis. Our data show that high DWV loads in winter bees correlate with reduced expression of genes involved in the cellular immune response and physiological activity and high expression of humoral immune genes involved in antibacterial defense compared with summer bees. This expression pattern could reflect evolutionary adaptations to resist bacterial pathogens and economize energy during the winter under a pathogen landscape with reduced risk of pathogenic viral infections. The outbreak of *Varroa destructor* infestation could have overcome these adaptations by promoting the transmission of viruses. Our results suggest that reduced cellular immune function during the winter may have increased honey bee’s susceptibility to DWV. These results contribute to our understanding of honey bee colony losses in temperate regions.

## Introduction

The western honey bee, *Apis mellifera*, must adapt to strong seasonal variations in climate and availability of food resources to survive extreme conditions. Consequently, two different types of bees can be found those produced for summer and those produced for winter. While the short-lived summer bees last only about one month, the winter bees can live up to six months [1,2].

Summer workers are generally reared, depending on the environmental situation, from March to July in the northern hemisphere. Workers perform different tasks in an age-dependent sequence: young workers usually care for the brood during the first two or three weeks of adult life while older workers forage for nectar and pollen outside the nest [3].

The long-lived winter bees begin to be produced in August and September in temperate climate conditions until brood rearing ceases, usually at the end of October. To overwinter, they form the winter cluster enabling thermoregulation of colonies [4]. From February on, they start to show a division of labor, similar to summer bees, to rear the new generation of summer worker bees [5].

Honey bees are permanently challenged by a large spectrum of pathogens, including ecto- and endoparasites, viruses and bacteria [6,7]. Although insects lack an acquired immune system to defend themselves against these pathogens, they have a well-developed innate immune system, which can be further divided into humoral and cellular responses [8,9].

The humoral immunity is mediated by four immune pathways: TOLL, IMD, JNK and JAK/STAT, which show diverse reactions against different intruders [9]. TOLL and IMD are two NF-kB-like signaling pathways involved in the control of genes coding antimicrobial peptides. Dorsal is a member of the NF-kB protein family involved in the regulation of the expression of *Defensin-1* and *hymenoptaecin* that act as antimicrobial effectors [9].

The cellular immunity is mediated by haemocytes and their response includes phagocytosis, nodulation and encapsulation [10]. Nodulation and encapsulation are often accompanied by melanization, [11] a process catalyzed by the *pro-phenoloxidase* (*PPO*) and *pro*-*phenoloxidase activator* (*PPOact*) genes [12–14]. Eater is a transmembrane protein involved in phagocytosis [15].

Interactions between *vitellogenin* (*Vg*) and juvenile hormone (JH) regulate important physiological processes in adult bees, including reproduction, division of labor and longevity [16–20]. Vg is a yolk protein expressed in the fat bodies, [18] secreted into the haemolymph, and then imported by developing oocytes through receptor-mediated endocytosis [21]. JH, one of the major insect hormones, is a sesquiterpene synthesized in the corpora allata [22]. In worker honey bees negative correlations between Vg and JH titers are associated with task performance. Under typical conditions, Vg levels are high during the first 2 weeks of workers adult life when performing tasks in the hive such as brood care and are low in older foragers [23]. JH titers follow an opposite pattern. Although the timing of nurse to forager transition depends on multiple factors, Vg and JH titers are in general predictable indicators of workers’ physiological states associated with behavioral development. Seasonal differences in Vg and JH titers also presumably reflect physiological differences between summer and winter bees. While JH titers have been found to be consistently higher in summer, compared with late fall and winter bees, Vg protein titers have been reported to be higher in late fall and beginning of the winter and lower at the end of winter [24,25].

Methyl farnesoate epoxidase (*mfe*) encodes the enzyme that catalyzes the oxidation of methyl farnesoate into JH III [26,27], the last step in the JH biosynthesis in most insects [28]. Recently Bomtorin et al., demonstrated that the expression of *mfe* in the corpora allata of adult worker honey bees corresponds with haemolymph JH titers and is consistently highly expressed in foragers compared with nurses [29]. These results indicate that quantification of *mfe* expression levels is a valid alternative to direct JH titer measurements.

Beekeepers in the Northern Hemisphere face considerable losses during the winter. One of the main causes is the infestation of the ectoparasitic mite *Varroa destructor* [30–32]. In addition to the direct effect on honey bee health, *V*. *destructor* mites are also an effective vector for viruses [33–36]. One virus that seems to play a crucial role in colony losses is the widespread deformed wing virus (DWV) [33,37,38]. DWV, a member of the Iflaviridae, can be found in all life stages of honey bees [39]. As its name indicates, causes deformed wings as well as other symptoms including shortened abdomens and premature death. Although not necessarily showing any malformations on adult honey bees, DWV might still reduce longevity [40] and the expression of immune genes [41], the latter leading to a potentially higher vulnerability to other pathogens. The combination of DWV infection and unfavorable environmental conditions may be a major contributor to winter losses.

Most of the colony losses occur during winter [30,31]. However most studies on honey bee immunity have focused on summer bees [42,43] and the immunity of winter bees is poorly understood [44]. The first aim of this study was to determine whether there are differences in the immune system between summer and winter bees. We hypothesize that higher incidence of colony losses during winter are associated with decreased immune function. To accomplish this objective, we measured the expression of immune genes involved in both the humoral and cellular immune responses from workers in field conditions. Our second objective was to test whether potential seasonal changes in immune gene expression are associated with different susceptibility to pathogens. To test this hypothesis, we selected the DWV, which is an excellent model of natural infection due its ubiquitous presence in *A*. *mellifera* colonies. Finally, our third objective was to investigate whether worker seasonal variation in immune gene expression and DWV load are associated with different physiological activity. For this purpose, we measured the expression of *Vg* and *mfe*, two genes associated with major seasonal and behavioral physiological differences in honey bees [24,29,45].

Summer and winter workers have evolved different physiology to cope with extreme differences in environmental conditions. Our results show that high loads of DWV in winter are associated with reduced expression of genes involved in the cellular immune response and physiological activity. Our findings are consistent with the hypothesis that down regulation of the energetically costly immune system and physiological activity under adverse winter conditions may be a strategy to save energy and increase overwintering survival even at the expense to increased risk of virus infection. Our results suggest a mechanism by which increased DWV infection, promoted by widespread *V*. *destructor* infestation, is associated with winter colony losses.

## Material and Methods

### 1. Experimental set up

Our experimental set up was completed in three main stages. First, establishment of source colonies: On June 1, 2012, six broodless colonies were established by using mated queens and 2 kg of young workers (predominantly *Apis mellifera carnica*) collected from 10 source colonies of our own local bee stock at the Swiss Bee Research Centre in Bern. Second, introduction of focal bees for summer collections: On June 5, 2012, a group of 500 newly emerged workers was introduced into 3 host colonies. Third, introduction of focal bees for winter collections: In September 11, 2012, a second a group of 500 newly emerged bees was introduced into the same host colonies.

For both groups of focal bees (summer and winter bees), capped brood from three different unrelated queenright source colonies were collected and transferred to an incubator at 34°C. Newly emerged workers were mixed together and marked. This experimental design randomizes the genetic background of the colonies studied: Although the genetic variation within colonies is high, variation across the different colonies is expected to be low.

Summer bees (N = 67) were collected weekly from June 5 to July 31, 2012 at 7 different time points. Ten workers were collected at each time point, with the exception of the last two points where were collected 8 and 9 workers, respectively. Winter bees (N = 50) were collected monthly from September 11, 2012 to January 13, 2013 at 5 different time points (N = 10 for each time point collection). Although two of these collection were performed during the fall (September and October) honey bee colonies have already transitioned to the winter physiological state (*diutinus* phenotype) by this time of the year in central European climate conditions [46]. The first collection in both groups (summer and winter bees) was performed using newly emerged bees. During winter, two hives needed to be combined before the last collection due to their low population. In all the samples, marked bees were taken alive from the hive, immediately frozen in liquid nitrogen and stored at -80°C until further processing.

*Varroa destructor* is ubiquitous in Switzerland and increased infestation levels of this ecto-parasite during fall and winter are associated with colony losses [35,47]. In order to reduce *V*. *destructor* infestation levels and ensure overwintering survival, hives were treated against *V*. *destructor* twice with formic acid in August/September and once with oxalic acid in December. Mite fall method was used to estimate *V*. *destructor* infestation through the experiment [48].

### 2. Real-Time qRT-PCR

RNA was extracted using the NucleoSpin RNA II Kit (Macherey-Nagel) and eluted with 75 μl of water. For each individual worker, cDNAs were synthesized using total RNA, M-MLV Reverse Transcriptase (Invitrogen), random hexamers (2.5 uM), oligo dT (0.1 μM) and dNTP (1.0 mM) in a final reaction volume of 20 μL. The thermal profile for cDNA synthesis was as follows: 25°C (10 min), 37°C (50 min) and 70°C (10 min). Afterwards the cDNA was diluted 1:10 with molecular grade H_2_O.

All samples were analyzed together using Hot start Kapa SYBR Fast qPCR. The expression of the genes *defensin-1*, *dorsal*, *eater*, *mfe*, *hymenoptaecin*, *prophenoloxidase*, *prophenoloxidase activator* and *Vg* as well as DWV loads was tested with specific primers, see Table 1. To normalize the data according to the total amount of RNA in each sample an analysis of the consistently expressed *β-actin* gene was performed [49]. Reactions preparations were performed as follow: 2 μl of the templates were mixed with 0.5 μl of forward and reverse primers (10μM), 6 μl of the supplier’s master mix (buffer and enzyme) and 3 μl H_2_O. For all the qPCR reactions the thermal profile used was the following: Polymerase activation (Hotstart) 95°C (3 min), 40 cycles of 95°C (30 sec) and 60° (20 sec).

**Table 1:** Primers used for qPCR.

Locus	F-primer	R-primer	Reference
DWV	GGA TGT TAT CTC CTG CGT GGA A	CCT CAT TAA CTG TGT CGT TGA TAA TTG	[66]
*defensin-1*	TGC GCT GCT AAC TGT CTC AG	AAT GGC ACT TAA CCG AAA CG	[67]
*dorsal*	TCG GAT GGT GCT ACG AGC GA	AGC ATG CTT CTC AGC TTC TGC CT	[41]
*eater*	CAT TTG CCA ACC TGT TTG T	ATC CAT TGG TGC AAT TTG G	[68]
*mfe*	GTT ATC GCT TCT GAT ATG GCT	GAT GGG AAA TAG GTA CCG AC	Corona unpublished
*hymenoptaecin*	CTC TTC TGT GCC GTT GCA TA	GCG TCT CCT GTC ATT CCAT T	[67]
*PPO*	CGC AAC TTA GAT GAA AAT AGA CC	TTG AGG CAT CCT TAC AAC CA	Corona, unpublished
*PPOact*	GCG TCC TCA TCA CGG ATA GAC A	AAA TCG TAT TCG CCG AGC C	Corona, unpublished
*Vg*	AGT TCC GAC CGA CGA CG	TTC CCT CCC ACG GAG TCC	[18]
*β-actin*	CGT GCC GAT AGT ATT CTT G	CTT CGT CAC CAA CAT AGG	[69]

### 3. Data analysis

Results from qPCR were converted with the software linReg PCR into N0 values. N0 is the starting concentration (N0) of the target per sample and calculated using the formula: N0 = Nq / (Emean^Cq) ([50,51]. The following formula was used to normalize the data: N0 _measured parameter_ / N0_*β-actin*_ = Relative quantities.

Since the data did not follow a normal distribution, the variation estimates of transcript between different groups were evaluated by using non-parametric Kruskal–Wallis and Mann-Whitney U tests as appropriate. Pairwise comparisons were performed with the Dwass-Steel-Chritchlow-Fligner Test. Multivariate Spearman correlations were performed between the variables (DWV and gene expressions). Spearman correlations were also used to estimate relation between aging of the workers and DWV loads. P-values below 0.05 were considered significant. The analyses were performed using Systat 13 software. The P-value significance for the Spearman correlations was calculated using the VassarStats program (http://vassarstats.net).

## Results

### 1. *Varroa destructor* treatments

At the end of July, the average daily mite fall/hive was 2 (± 1.6 Standard Error). In December, before the treatment with oxalic acid, daily mite fall increased to 14 ± 3.5. After this treatment, mite fall was reduced to 3 ± 0.4. Daily mite fall numbers immediately after the treatments were not included in the winter counts.

### 2. Immune gene expression

**Fig 1:**
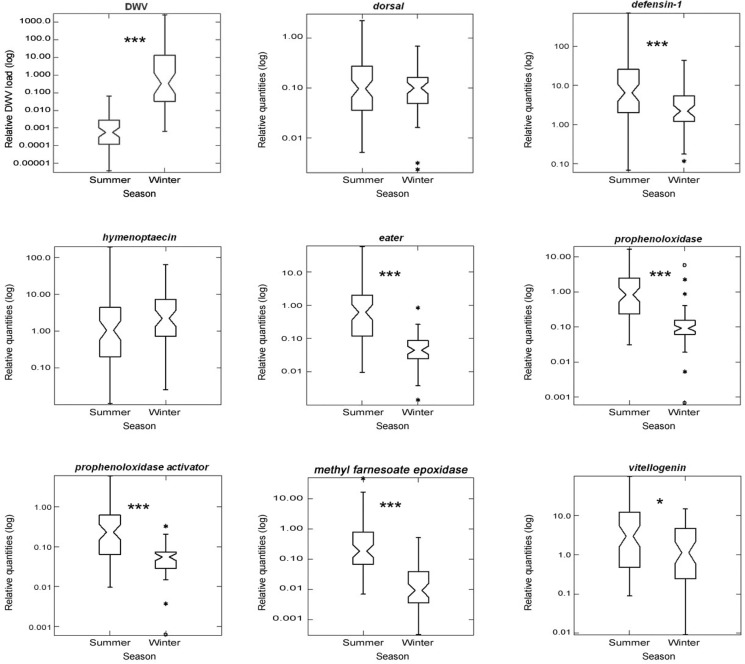
Variability of DWV load, expression of immune and physiological genes in summer (N = 67) and winter bees (N = 50) according to age. The Y-axis show the relative DWV genome copies per bee (log10) or the different genes expressions. The X-axis shows the age of the bees for both summer and winter samples. (Kruskal-Wallis Analysis of Variance, significant differences are indicated with *P<0.05; ***P<0.001). Gene expression profiles in summer and winter bees are indicated with continuous and dotted lines, respectively.

**Fig 2:**
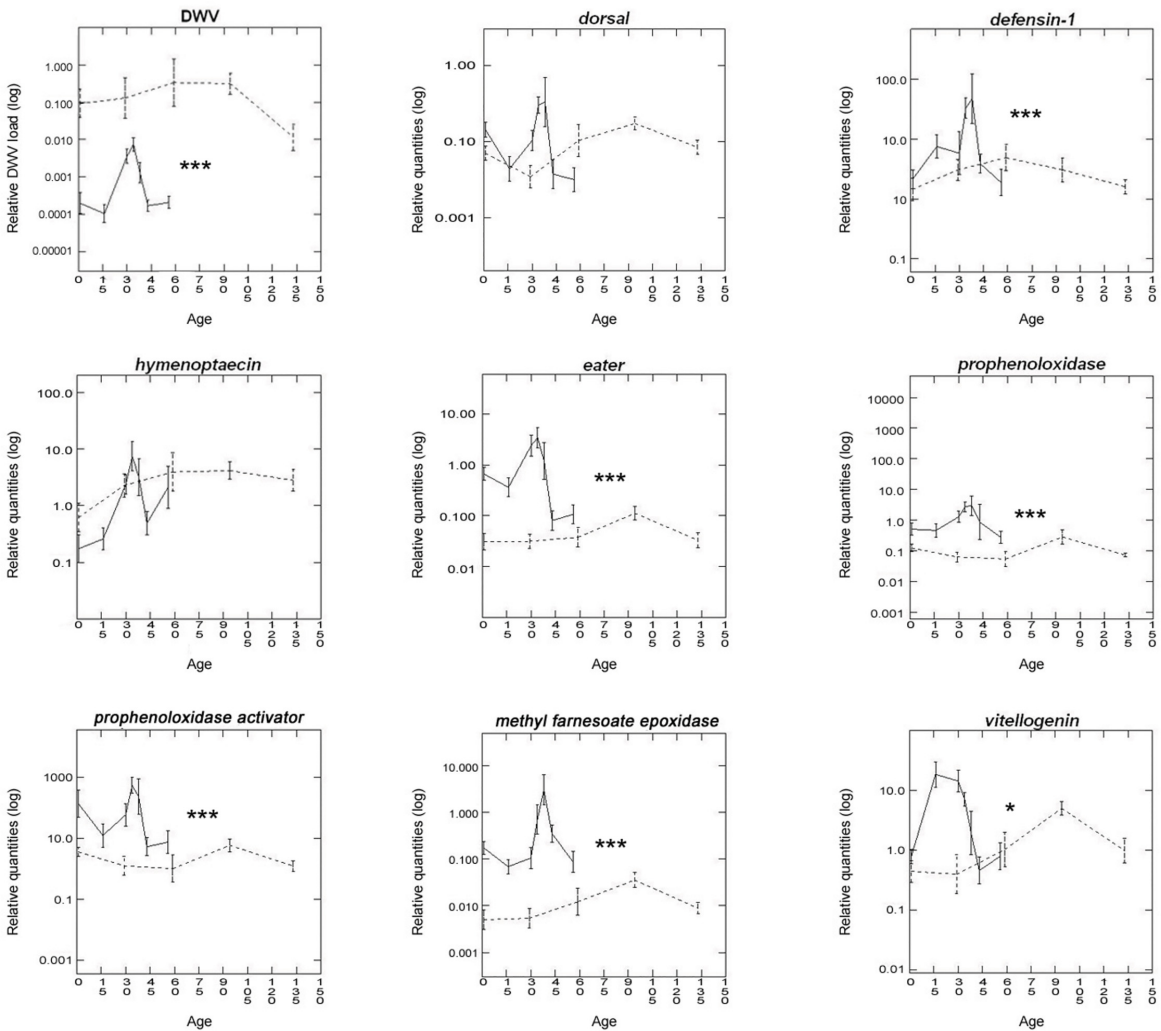
Seasonal variability in gene expression profiles and DWV load. The Y-axis shows the relative quantities of the measured genes, respectively relative genome copies of DWV (log10). The two groups are shown in the X-axis, summer bees (N = 67) winter bees (N = 50). (Mann Whitney U test, significant differences are indicated with *P<0.05; ***P<0.001).

Most of the immune genes analyzed showed lower expression in winter bees compared with summer bees (Mann-Whitney test, P<0.001). *Dorsal* and *hymenoptaecin* were the only immune genes that did not show seasonal differences in expression (Mann-Whitney test, P = 0.116, resp. P = 0.509). In contrast, *eater*, *PPO* and *PPOa* showed the highest changes in expression (P<0.001) (Fig 1). Differences in the expression of these genes were apparent since the first collection of newly emerged bees (Mann-Whitney test, P<0.05) (Fig 2). In summer, all immune genes analyzed showed a peak in their expression when the bees were 30–40 days old. On the other hand, in winter, while *defensin-1* and *hymenoptaecin* reached their highest expression levels in 60-day old bees, *dorsal* and the genes involved in the cellular immune response (*eater*, *PPO* and *PPOa)* showed their highest expression levels when the bees were 90-days old (Fig 2, Table 2).

**Table 2:** Significant variations between single ages in pairwise comparisons.

	Summer		Winter	
Gene	Days Compared	P-value	Days Compared	P-value
***defensin-1***	1 vs 30	<0.05		
***defensin-1***	1 vs 34	<0.01		
***defensin-1***	16 vs 34	<0.05		
***defensin-1***	34 vs 43	<0.05		
***dorsal***	34 vs 43	<0.05		
***dorsal***			29 vs 94	<0.05
***eater***	34 vs 43	<0.05		
***eater***			94 vs 133	<0.05
***hymenoptaecin***	1 vs 30	<0.05		
***hymenoptaecin***	1 vs 34	<0.01		
***hymenoptaecin***	16 vs 30	<0.01		
***hymenoptaecin***	16 vs 34	<0.01		
***hymenoptaecin***	16 vs 56	<0.05		
***hymenoptaecin***	34 vs 38	<0.05		
***hymenoptaecin***	34 vs 43	<0.05		
***PPO***	16 vs 34	<0.01		
***PPOact***	1 vs 16	<0.05		
***PPOact***	16 vs 34	<0.01		
***PPOact***	34 vs 43	<0.05		
***mfe***	1 vs 43	<0.01		
***mfe***	16 vs 34	<0.001		
***mfe***	16 vs 43	<0.05		
***mfe***	30 vs 43	<0.01		
***mfe***	43 vs 56	<0.05		
***Vg***	1 vs 30	<0.05		
***Vg***	1 vs 34	<0.05		
***Vg***	16 vs 38	<0.05		
***Vg***	30 vs 38	<0.01		
***Vg***	30 vs 43	<0.05		
***Vg***	30 vs 56	<0.05		
***Vg***	34 vs 43	<0.05		
***Vg***			1 vs 94	<0.01
**DWV**	1 vs 30	<0.01		
**DWV**	1 vs 34	<0.01		
**DWV**	16 vs 30	<0.001		
**DWV**	16 vs 34	<0.001		
**DWV**	30 vs 38	<0.01		
**DWV**	30 vs 43	<0.01		
**DWV**	34 vs 38	<0.01		
**DWV**	34 vs 43	<0.01		
**DWV**			59 vs 133	<0.05

Summer bees (N = 67), winter bees (N = 50).

### 3. DWV load

The prevalence of DWV was very high in the tested population, with 95.5% and 100% of workers infected in summer and winter bees, respectively. Winter bees had a significantly higher number of DWV copies per bee than summer bees (Mann-Whitney test, P<0.001). The newly emerged winter bees had an average DWV load more than 300-fold higher than summer bees of the same age (Fig 2). Both groups showed highly significant variations between their respective lowest and highest DWV load (Dwass-Steel-Chritchlow-Fligner, P<0.001).

No significant correlations between age and DWV load were observed either in summer (Spearman, r_s_: 0.063, P = 0.306) or winter (Spearman, r_s_: -0.231, P = 0.053). Instead, both groups showed an initial increase followed by a decrease of DWV genome copies per individual (Fig 2).

### 4. Genes associated to physiological status

When comparing summer and winter bees, *mfe* showed strong differences in expression in total, and at the first day (Mann-Whitney test, P<0.001). *Vg* levels showed no differences in freshly emerged bees (Mann-Whitney test, P = 0.290) whereas it did at the later stage (Mann-Whitney test, P<0.05) (Figs 1 and 2). In summer, *mfe* showed low levels during the first 30 days, increased to its maximum point at 45 days, and declined afterwards. Conversely, *Vg* levels increased to their highest point between 15–30 days and then quickly declined in older workers. In winter, contrary to summer, both genes showed a similar expression pattern: mRNA levels began low during the first 30 days, and then increased until reaching their highest levels around 90 days.

### 5. Correlations between DWV load and the expression of genes involved in immune response and physiological status

**Fig 3:**
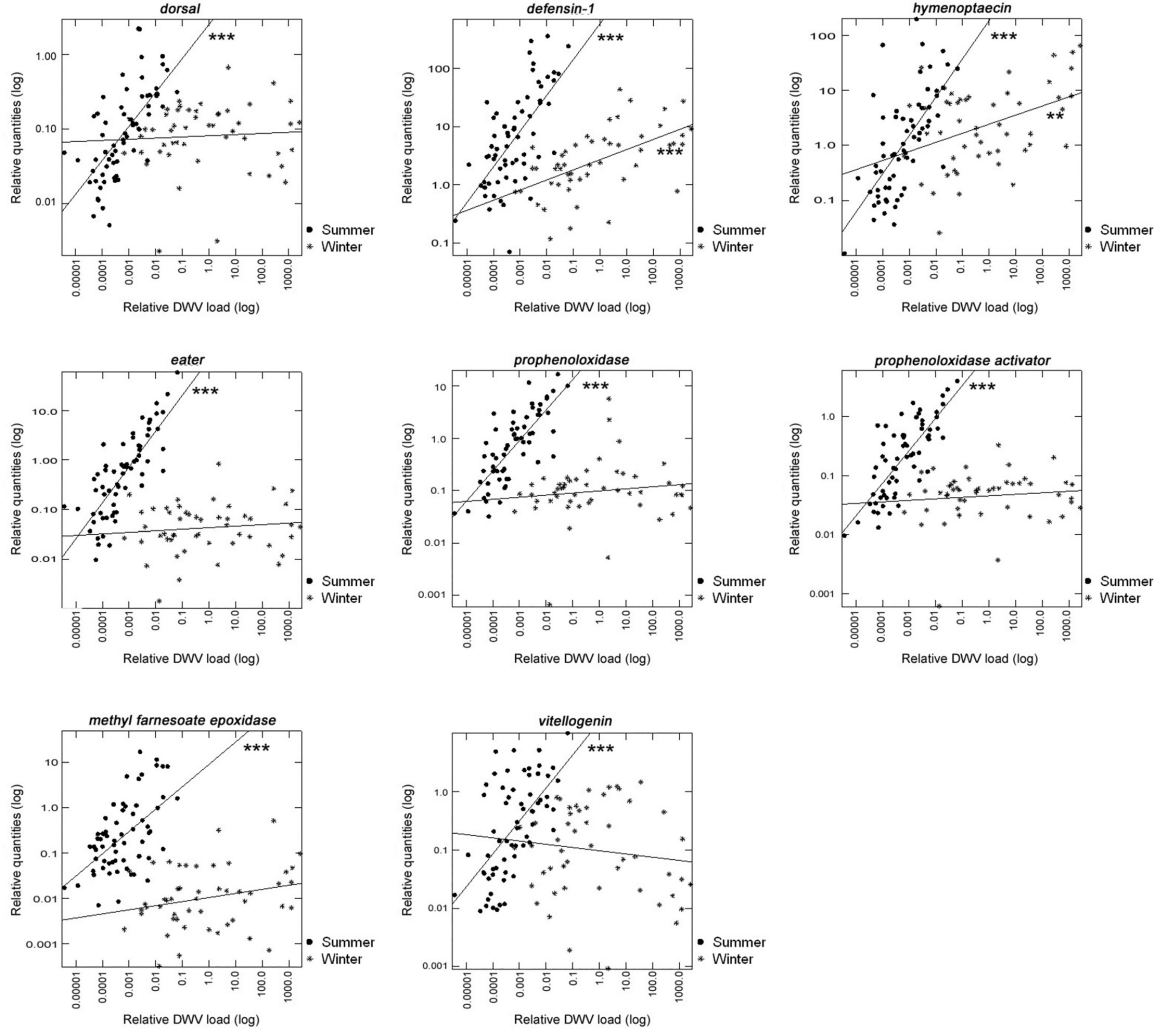
Correlations of relative gene expression to DWV load. Y-axis show relative quantities of gene expressions (log10). X-axis show relative DWV genome copies (log10). (●) Summer (N = 67), (*) winter (N = 50). (Mann-Whitney test, **P<0.01; ***P<0.001).

During the summer observation period the expression of genes involved in both immune response and physiological activity strongly correlated with DWV load (Spearman, r_s_: 0.417–0.722, P<0.001). In contrast, winter bees showed no correlation between the expression of these genes and increasing DWV load, with the exception of *defensin-1* and *hymenoptaecin* (Fig 3 and Table 3). In summer bees, the peak of DWV load occurred when the bees were approximately 30 days old, a period characterized by decreasing *Vg* levels and increasing expression of *mfe* and immune genes. In winter, DWV levels peaked when the bees were 60–90 days old and declined afterward, correlating with the peak of expression of dorsal and the cellular immune genes (Fig 2).

**Table 3:** Spearmans r_s_ correlation of genes expressions with DWV load on summer and winter bees.

Gene	summer bees (N = 67)		winter bees (N = 50)	
	Spearman r_s_	P-value	Spearman r_s_	P-value
***defensin-1***	0.722	<0.001	0.567	<0.001
***Dorsal***	0.604	<0.001	0.060	0.339
***Eater***	0.741	<0.001	0.070	0.315
***hymenoptaecin***	0.711	<0.001	0.351	**<0.01**
***PPO***	0.648	<0.001	0.138	0.170
***PPOact***	0.603	<0.001	0.127	0.189
***mfe***	0.417	<0.001	0.247	0.083
***Vg***	0.608	<0.001	-0.076	0.300

P values <0.05 were considered significant.

### 6. Age-related expression of immune and physiological genes

The expression of all the tested genes associated with immune response and physiological activity was significantly lower in winter bees compared with summer bees (Mann-Whitney test, P<0.05) (Fig 1). During the summer, most of these genes showed no overall correlation with age, with the exception of *hymenoptaecin* and *mfe*, which showed a positive correlation. In winter bees, the genes that showed age-related correlation in their expression were *Vg* and *hymenoptaecin*, which were positively correlated with age, and *PPO*, which showed a negative correlation. *Hymenoptaecin* was the only gene that showed an overall positive correlation with age in both seasons (Fig 2 and Table 4). Interestingly, in summer bees, the period of higher Vg expression (15–30 days) precedes the peak of expression of both types of immune genes and *mfe* (30–40 days) (Fig 2).

**Table 4:** Spearmans r_s_ correlation of gene expression with worker age on summer and winter bees.

Gene	summer bees (N = 67)		winter bees (N = 50)	
	Spearman r_s_	P-value	Spearman r_s_	P-value
**DWV**	0.063	0.306	-0.231	0.053
***defensin-1***	0.158	0.101	-0.074	0.304
***dorsal***	-0.082	0.255	0.210	0.072
***eater***	-0.025	0.420	0.065	0.327
***hymenoptaecin***	0.358	<0.01	0.273	<0.05
***PPO***	0.042	0.368	-0.262	<0.05
***PPO activator***	-0.103	0.203	-0.209	0.073
***mfe***	0.392	<0.001	0.180	0.228
***Vg***	-0.074	0.276	0.383	<0.01

P values <0.05 were considered significant.

### 7. October exception

Samples collected in October showed disparate results. In contrast with other winter collections, some of the genes analyzed in the samples collected during this month showed a negative correlation with DWV load. This correlation was significant only in the cases of *dorsal*, *PPO* and *PPOact*. An exception to this trend was the expression of *defensin-1*, which had a positive correlation with DWV, although not significant (Table 5).

**Table 5:** Spearmans r_s_ correlation of genes expressions with DWV load in samples collected in October (winter bees).

Gene	October samples (N = 10)	
	Spearman r_s_	P-value
***defensin-1***	0.018	0.48
***dorsal***	-0.648	<0.05
***eater***	-0.176	0.313
***hymenoptaecin***	-0.164	0.325
***PPO***	-0.564	<0.05
***PPO activator***	-0.552	<0.05
***mfe***	-0.176	0.313
***Vg***	-0.527	0.059

P values <0.05 were considered significant.

## Discussion

We hypothesized that a physiological adaptation of winter bees to increase winter survival is associated with an overall decrease in physiological activity, including down regulation of the energetically expensive immune system, which results in increased susceptibility to pathogens. To test this hypothesis we measured the expression of genes involved in immune response, physiological activity, and the naturally acquired load of DWV in summer and winter bees.

Our results show that winter workers exhibit reduced expression of immune genes and higher DWV loads compared with summer bees. We also showed interactions between immune response and the expression of genes associated with physiological activity. The expression of *Vg* and *mfe* was reduced in winter bees, suggesting that overall reduced metabolic activity is concomitant with decreased immune function and higher susceptibility to DWV infection.

Consistent with previous studies [33,38,40], we found that winter bees have a much higher DWV load per individual compared to summer bees. Overall, DWV loads were not correlated with age in the summer or winter bees. However, they experienced an initial increase of DWV load, followed by a subsequent decrease. These results can be explained by two different hypotheses. First, bees with higher DWV load died while workers with lower loads survived [40]. Second, an antiviral immune response targeting DWV results in lower DWV loads in older bees. Our data did not provide us with the information needed to distinguish between these two alternative hypotheses.

From summer to winter, the *V*. *destructor* count increased in daily mite fall. During this period the DWV load in newly emerged bees increased by an average factor of 300. These results are consistent with previous reports showing that increased mite infestation is associated with higher DWV infection [33,38,40].

Most of the immune genes analyzed in our study showed significantly lower expression in winter bees compared with summer bees. Winter bees in particular showed a very low expression of *eater*, *PPO* and *PPOa*. In contrast, *Dorsal* and *hymenoptaecin* were the only immune genes that did not show seasonal differences. Interestingly, although *defensin-1* was highly expressed in the summer, it was still the highest expressed immune gene in winter bees.

During the summer all of the tested immune genes showed positive correlation with DWV load. In contrast, winter bees showed no correlation between immune gene expression and DWV, with the exception of *defensin-1* and *hymenoptaecin*, which showed a positive correlation with increased DWV load. These results show that the immune genes most strongly down regulated in winter showed no correlation with DWV and that the genes that did not show reduced expression during the winter were in general positively correlated with DWV load. Interestingly, the genes strongly down regulated in the winter that did not show correlation with DWV include *eater*, *PPO* and *PPOa*, which belong to the cellular immune response, while the genes that do not show reduced expression during the winter and were positively associated with DWV, belong to the TOLL-IMD pathways involved in the humoral response to bacterial infection. Altogether, these results show that while most of the immune system is down regulated, genes coding antibacterial effectors are actively expressed in winter bees. This expression pattern suggests that bacterial infection may be an underlying factor promoting DWV replication. Indeed, bacterial challenge induces DWV replication [52] and reduces bee survival [53]. It has been proposed that bacterial infection can be a consequence of the wounds produced by *V*. *destructor* feeding [52]. Additionally, winter bees, which rarely leave the hive for defecation, present ideal conditions for incubation and growth of gut pathogens. Our results, together with previous studies showing increased expression of genes coding antimicrobial peptides in winter bees [54], suggest that winter bees experience bacterial infections. However, it remains to be verified if this is the case and whether bacterial infection promotes DWV replication during the winter.

In addition to *PPO* and *PPOa*, other genes coding enzymes involved in the cellular response, including glucose dehydrogenase (GLD), have been found to be negatively correlated with DWV [52]. GLD has been proposed to be required for neutralization of pathogens during the encapsulation reaction via oxidative free radicals and reacts with the quinones produced by PO [55]. Although the PO-mediated melanization reaction system has not yet been shown to be involved in antiviral resistance in honey bees, evidence obtained from other insects suggests that the reduced expression of genes involved in PO pathway may be related to decreased antiviral resistance. First, PO activity has a virucidal effect in the tobacco budworm *Heliothis virescens* [56]. Second, it has been found that polydnaviruses encode for proteins that inhibit the PO activation pathway in *Manduca sexta*, suggesting that interfering the melanization response is a strategy to evade insect immune defenses [57].

Studies in newly emerged bees have showed a negative correlation between DWV load and the expression of immune genes, especially those involved in the cellular immune response [52,53]. While this pattern strongly contrasts with our results in the spring, it partially resembles our results in winter where only the genes involved in the humoral response to bacterial infection were positively associated with DWV and the genes involved in cellular immune response have very low levels compared with summer bees.

Immuno-suppression associated with DWV also has been reported in bees collected in the fall, where the expression of regulatory genes such as dorsal was particularly reduced in bees with high DWV loads [41]. Remarkably, the same study demonstrated that dorsal RNAi-mediated knockdown result in increased DWV. While this study suggests that decreased immune function promotes DWV replication, the possibility of an immune-suppression effect caused by high DWV levels cannot be discarded.

Low levels of DWV can be detected even in newly emerged bees, which were not infested with *V*. *destructor*, indicating that this virus may be transmitted through a vertical transmission pathway [37,58]. Honey bees experience increased DWV load after *V*. *destructor* infestation, independently of whether they come from colonies with higher or lower DWV loads, suggesting that *V*. *destructor* is not only a horizontal vector of DWV but also actively promotes its replication [33,36,59]. Several lines of evidence suggest that increased DWV replication is the result of decreased immune function caused by the interplay of *V*. *destructor* infestation and seasonal changes in environmental conditions that alter the host nutritional-energetic balance. First, *V*. *destructor* feeding presumably results in malnutrition by draining the host haemolymph proteins [17]. Second, harsh pre-winter environmental conditions such as decreased temperatures and reduced access to nutritional resources [60] can compromise the nutritional state of honey bee colonies, especially those that have not accumulated enough nutritional reserves. Third, strong colonies in the fall, with increased colony-level (honey and pollen) and individual level (store protein such as Vg) reserves are more likely to successfully overwinter compared with weak colonies [33,38]. These results suggest that critical adaptations to winter survival include both increased synthesis of stored proteins and reduced expression of immune genes. Weak colonies may not only fail to build protein reserves but experience further decrease in the expression of immune genes. We hypothesize that while detrimental effects of *V*. *destructor* infestation promoting DWV replication occur during the whole life cycle of honey bees [17], its effect becomes critical in fall and winter when increasing mite infestation levels are concomitant with a seasonal decline in immune function. Consistent with this hypothesis, during the summer the expression of immune genes is positively associated with DWV, suggesting not only that under these conditions (e.g., absence of nutritional stress) DWV does not have an immune suppression effect, but there is also an active immune response reacting to its presence and possibly involved in its clearance.

In this study, we measured the expression of Vg and a gene coding for the last enzyme involved in JH synthesis (*mfe*), which have been shown to correlate with JH haemolymph titers [29]. Our results showed in general lower levels of *Vg* and *mfe* in winter bees compared with summer bees, which is consistent with previous studies where Vg and JH titer were determined [24,25]. However, there were some interesting exceptions:

First, contrasting with previous reports, we observed low *Vg* mRNA levels during the fall. In typical conditions, the queen reduces egg laying during the fall and most of the in-hive colony population is composed of over-aged nurses. In contrast, we started our fall collections with newly emerged bees. Pre-winter environmental signals such as increasingly low temperatures, reduced nutritional resources and decreased brood rearing [60] correlate with increased Vg levels in fall [24]. We hypothesize that these environmental signals leading to increased Vg expression may have been missed by the newly emerged bees introduced into host colonies during the fall, resulting in lower Vg expression in these bees. These results support the hypothesis that the potential for building increased Vg reserves for overwintering is affected by pre-winter colony conditions. Consistently, Dainat et al., 2012 [38] found that higher Vg mRNA levels in fall were only found in colonies that successfully overwintered, revealing an association between Vg expression and colony survival.

Second, in summer bees we found highly-sustained *Vg* expression levels until the first 30 days followed by an increase in *mfe* expression afterward. This pattern of alternated Vg and *mfe* expression associated with the nurse to forager transition is considerably delayed compared with previous studies where Vg and JH titers were determined [24,25]. In contrast with these studies, we introduced newly emerged bees into broodless colonies. Bees under this condition are known to experience high Vg titers, delayed nurse to forager transition and extended life span. This pattern is consistent with our results, where in addition to the delayed switch between *Vg* and *mfe* expression, we observed an unusual longevity in the bees collected during the summer: we were able to collect bees up to an age of 56 day, which almost double the normal lifespan expectancy of summer bees [5]. Overall, the use of *Vg* and *mfe* as molecular markers of physiology proved to be a useful method to assess the physiological activity associated with seasonal and behavioral states.

Our results obtained in October deserve special attention. Samples collected during this month were the only group that showed a negative correlation between DWV and all tested genes with the exception of defensin-1. Interestingly, this negative correlation was significant only in the case of dorsal, PPO and PPOa, immune genes which expression has been shown to be particularly negatively associated with DWV load in previous studies [38,41,52]. These results further support the hypothesis that fall represents a critical period when honey bee colonies experience important nutritionally-dependent physiological adaptations to survive winter and that food reserves and *V*. *destructor* infestation levels are key factors that determine the capability of colonies to undergo these adaptations [17,45]. Our results obtained in October seem to mimic the conditions of weak colonies with reduced Vg levels where the effect of *V*. *destructor* promoting DWV replication is enhanced by further reduction in immune function.

## Conclusive Remarks

During the past 50 years, the global spread of the ectoparasitic mite *Varroa destructor* has resulted in the death of millions of honey bee colonies [61]. There is a general consensus that the mites association with honey bee viruses is an important contributing factor in the global collapse of honey bee colonies. However, most honey bee viral infections were considered harmless before the spread of *V*. *destructor* [31].

Our results show that, compared with summer bees, winter bees exhibit reduced expression of genes involved in the cellular immune response and physiological activity, while maintain high expression of humoral immune genes involved in antibacterial defense. We propose two mutually non-exclusive hypotheses to explain these results:

First, adaptive advantages of decreased energetically costly immune function and physiological activity during the winter could provide a mechanism to economize energy under extreme environmental conditions, even at the expense of risk of viral infection. Under this hypothesis, the balance between overwintering survival (promoted by energy saving) and mortality (by increased risk of virus infection) may have been shifted by the spread of *V*. *destructor*. Second, the observed expression of immune genes in winter could reflect past evolutionary adaptations to existing pathogens commonly affecting honey bee colonies or even co-evolution with its own bacteriome. Before the outbreak of *V*. *destructor* infestation, the risk of bacterial infection during the winter (e.g., due to fermentation of bacterial flora in the intestines) [54], may have been more important than the risk of pathogenic virus infection. Thus, honey bees may have evolved an immune system better adapted to resist bacterial, rather than virus infections. The recent outbreak of *V*. *destructor* infestation, could have overcome this adaptation by promoting the replication and prevalence of pathogenic DWV strains [34,59,62], which could have exploited this immunological vulnerability. Previous studies showing no correlation between the presence of *V*. *destructor* and changes in host immune responses [63,64], support the hypothesis that the honey bee immune system has not been adapted yet to the recent upsurge of DWV infection. Although this last hypothesis specifically addressed the differential expression of immune genes observed in winter bees, the adaptive value of economizing energy cannot be ruled out, especially after the outbreak of *V*. *destructor* infestation, which further compromises colony nutritional reserves by different means, including direct feeding on haemolymph proteins. In any case, before the arrival of *V*. *destructor* in populations of European honeybees, virus outbreaks could also occur, e.g. the famous “Isle of Wight Disease”, which was probably caused by chronic bee paralysis virus [32]. DWV can also potentially act independently of *V*. *destructor* to bring about colony losses [65], which might be related to both quantitative and qualitative changes in the honeybee viral landscape after the arrival of this mite acting as a new and efficient vector [62].

This study shows important differences in the expression of genes involved in immune response and physiological activity between summer and winter bees, which contribute to explain the proximal and evolutionary mechanisms associated with honey bee winter colony losses. These results are in line with previous findings showing that *V*. *destructor* infestation is an important contributing factor associated with decreased honey bee populations [30,34,38,59].
